# The Role of Air Pollution Exposure and *GSTM1-/GSTT1*-Null Genotypes in Gestational Diabetes Mellitus Development: A Case–Control Study on Gene–Environment Interactions

**DOI:** 10.3390/antiox14060652

**Published:** 2025-05-28

**Authors:** Ana Susa, Dragana Davidovic, Nadja Nikolic, Tamara Sljivancanin Jakovljevic, Vera Kujundzic, Sladjana Mihajlovic, Ljiljana Bogdanovic

**Affiliations:** 1 Faculty of Medicine, University of Belgrade, 11000 Belgrade, Serbia; 2 Institute of Hygiene and Medical Ecology, 11000 Belgrade, Serbia; 3 Implant-Research Centre, School of Dental Medicine, University of Belgrade, 11000 Belgrade, Serbia; 4 Department of Neonatology, The Obstetrics and Gynecology Clinic “Narodni Front”, 11000 Belgrade, Serbia; 5 University Hospital “Dr Dragiša Mišović–Dedinje”, 11000 Belgrade, Serbia; 6 Institute of Pathology, 11000 Belgrade, Serbia

**Keywords:** gestational diabetes mellitus, air pollution, glutathione transferase, particulate matter, ozone, gene–environment interaction

## Abstract

As gestational diabetes mellitus (GDM) rises as a major public health concern, various factors have been identified as potential contributors, with air pollution drawing increasing attention. The mechanisms by which air pollutants lead to detrimental impacts are largely attributed to oxidative stress. However, the role of air pollution is still not entirely clarified, suggesting that additional factors, such as genetic variability, particularly of genes involved in redox homeostasis, influence the GDM risk. This study addresses three questions: (1) whether ambient PM_2.5_, PM_10_, O_3_, and NO_2_ exposures associate with GDM risk; (2) if *GSTM1-/GSTT1*-null genotypes affect the risk of GDM; and (3) whether these genotypes modify pollution–GDM associations. This case–control study comprised 133 women in the case group and 144 in the control group. Exposure to air pollutants was assessed based on the participants’ residential addresses and during different time windows: pre-pregnancy period, first trimester, and second trimester. *GSTM1/GSTT1* genotyping was conducted from blood samples. Higher PM_2.5_, PM_10_, and O_3_ levels increased GDM risk in women. While *GSTM1-/GSTT1*-null genotypes showed no overall link to GDM, non-smokers with *GSTM1*-null had higher GDM risk when exposed to PM_2.5_ during the first trimester. While further research on gene–environment interactions is needed, our findings highlight that reducing air pollution may lower GDM risk.

## 1. Introduction

Gestational diabetes mellitus (GDM) is often defined as a glucose metabolism disorder with onset or first recognition during pregnancy [1]. Given the increasing trend in prevalence in recent years, as well as the severe complications that can occur if left untreated, GDM is recognized as a significant public health problem [2]. In Serbia, addressing the issue is further complicated by limited data on prevalence and trends of GDM, due to the absence of centralized registries and a severe lack of studies covering this topic. If we were to observe the prevalence of GDM in the region, the data bring even more concern, as Eastern (weighted average of 31.5%) and Southern (weighted average of 12.3%) Europe tend to have the highest prevalences of GDM in Europe [3].

Regarding health consequences, women with GDM have a higher probability of developing associated complications, such as gestational hypertension, pre-eclampsia, and polyhydramnios, and are at a higher risk of Caesarean delivery. Although it often resolves after delivery, women who were diagnosed with GDM are at an increased risk of developing type 2 diabetes mellitus (T2DM) later in life. Aside from maternal complications, newborns may also be affected, as fetal macrosomia, preterm birth, and birth trauma are more common in children of mothers with GDM. In addition to these short-term complications, these children are at higher risk of developing T2DM, obesity, and metabolic syndrome later in life [4].

Like other chronic non-communicable diseases, the development of GDM is dependent on a myriad of complex pathophysiological processes. Oxidative stress, one of the significant factors that trigger these processes, occurs when reactive oxygen species (ROS) exceed the body’s capacity to neutralize them. In GDM, oxidative stress acts both directly, through cellular damage, and indirectly, by inducing inflammation. Cellular damage could be directed towards pancreatic β-cells, whose insulin secretion becomes impaired. Inducing inflammation, on the other hand, affects the insulin signaling pathways, thereby promoting insulin resistance. Normal glucose regulation during pregnancy is therefore likely dependent on achieving redox homeostasis—a fine balance between the production of ROS and the body’s capacity for their removal [5].

In order to better understand this disorder, scientists around the world are investigating various factors that could potentially increase the risk of developing it. To this point, some of the better-known risk factors include advanced maternal age, the occurrence of GDM in past pregnancies, an unhealthy diet, and insufficient physical activity [6,7,8]. Newer studies point to the influence of other factors, predominantly genetic and environmental [9,10].

With the increased interest in climate change, air pollution has gained considerable attention in recent years. A significant number of studies have pointed to air pollution as an emerging risk factor for many non-communicable diseases, including respiratory [11,12], cardiovascular [13,14], neurological disorders [15], as well as various types of cancer [16,17,18]. The primary and most notable pathophysiological mechanism through which air pollutants impact health is largely attributed to the increased generation of ROS and the resulting oxidative stress [19,20]. Given the fundamental role of oxidative stress in the development of GDM, air pollution has been the focus of scientific research aimed at explaining the complex mechanisms underlying GDM development. However, the findings of these studies are somewhat ambiguous, with some results pointing to an increased risk of exposure to certain air pollutants and others showing no significant association or even inverse correlations [21,22,23]. This seemingly protective effect, observed through inverse association, particularly reported for ozone (O_3_), is entirely unclear. One possible explanation could be that high pollutant levels may induce early pregnancy loss, thereby excluding the most severely affected women. The confusing and inconsistent results observed across these studies may also be explained by employing different methodologies of exposure assessment, different lifestyles among populations, or the effects of genetic susceptibility.

Glutathione S-transferases (GSTs) are members of a superfamily of widespread enzymes whose main role is to protect cells against various toxic substances, both endogenous and exogenous (e.g., environmental pollutants). Their point of action is during the phase II detoxification process, where they catalyze the binding of reduced glutathione (GSH) to various hydrophobic electrophilic compounds. This reaction produces a water-soluble GSH–substrate complex, which is more easily eliminated. Each GST-mediated binding reaction requires a significant metabolic cost, primarily through GSH consumption. Furthermore, maintaining an adequate supply of reduced GSH requires NADPH expenditure. Consequently, sustained detoxification depletes both GSH and NADPH levels, compromising antioxidant defenses and amplifying oxidative stress [24].

The efficiency of GST-mediated detoxification is determined by transcriptional and genetic mechanisms. Under basal conditions, expression of genes encoding these enzymes, such as *GSM1* and *GSTT1*, remains low; however, oxidative stress triggers their upregulation via the Nrf2-Keap1/ARE pathway. Under stressed conditions, Keap1 becomes oxidized, which prevents it from binding to Nrf2, enabling its nuclear translocation and binding to antioxidant response elements (AREs) in *GSTM1* and *GSTT1* promoters [25]. However, the transcription would not be possible if the target genes were deleted, leading to the absence of GSTs [24]. Moreover, even in wild-type individuals, epigenetic silencing (e.g., promoter hypermethylation) or miRNA-mediated repression can attenuate the expression of target genes, thereby reducing the detoxification capacity [26].

Still, current epidemiological evidence regarding associations between *GSTM1* and *GSTT1* gene deletions and the risk of developing diseases related to oxidative stress remains inconsistent. Studies on GDM in particular are somewhat scarce and report conflicting results [27,28]. This discrepancy may stem from factors not typically covered in conventional genetic studies, such as lifestyle factors or environmental influences.

This study addresses three critical questions: (1) whether ambient PM_2.5_, PM_10_, O_3_, and NO_2_ exposures associate with GDM risk; (2) if *GSTM1-/GSTT1*-null genotypes affect the risk of GDM; and (3) whether these genotypes modify air pollution–GDM associations. Our findings provide evidence on environmental determinants of GDM while assessing the role of *GSTM1-/GSTT1*-null genotypes in polluted environments.

## 2. Materials and Methods

### 2.1. Study Design and Setting

This case–control study was conducted from September 2023 to May 2024 at the Hospital for Gynecology and Obstetrics, University Hospital “Dr Dragiša Mišović–Dedinje”, a secondary healthcare center located in Belgrade, Serbia.

The recruitment process, data and sample collection and storage, and analyses were conducted with respect to participants’ confidentiality and integrity and per the Declaration of Helsinki and applicable national legislation on biomedical research. The study was approved by the Ethical Committee of the University Hospital “Dr Dragiša Mišović–Dedinje” (no: 12890/2-2023, date: 20 June 2023), as well as the Ethical Committee of the Faculty of Medicine, University of Belgrade (no: 17/X-5, date: 24 October 2023). After receiving a comprehensive description of the study details, participants who agreed to take part in the study signed the Informed Consent.

### 2.2. Study Participants

The participant recruitment was conducted at the time of delivery. Cases included women who were diagnosed with GDM based on the American Diabetes Association guidelines. Following the 75 g Oral Glucose Tolerance Test (OGTT) between the 24th and 28th gestational week, women were considered eligible for the study if any of the following blood glucose limit values were exceeded: fasting blood glucose >5.1 mmol/L, 1 h blood glucose >10 mmol/L, or 2 h blood glucose >8.5 mmol/L [29]. To maintain the focus on a specific disorder, participants with preexisting diabetes mellitus, as well as GDM in previous pregnancies, were considered ineligible. Given an increased risk of developing GDM, women suffering from chronic kidney disease were also excluded from the study. Individuals receiving corticosteroids, as well as those with multiple pregnancies, were excluded from participation due to pre-existing disruptions in their glucose regulation. Lastly, women below the age of 18 and those who frequently relocated (more than three times) or lived abroad within the year preceding the delivery were also excluded from the study. The control group included healthy women with singleton pregnancies over the age of 18, permanently residing in Serbia. The study ultimately included a total of 277 pregnant women, with 133 women diagnosed with GDM forming the case group and 144 healthy women comprising the control group.

### 2.3. Data Collection

Information about the participants’ age and pregnancy-related clinical characteristics was collected from medical records and further verified through interviews with the women who participated in the study. During these interviews, participants also provided details regarding their residential address and mobility, average time spent outdoors during the period of interest, and smoking habits.

### 2.4. Air Pollution Exposure Assessment

To evaluate exposure, spatial interpolation was conducted utilizing publicly available data obtained from the Serbian Environmental Protection Agency’s open data portal [30]. These datasets included daily concentrations of particulate matter with aerodynamic diameter less than 2.5 μm (PM_2.5_) and less than 10 μm (PM_10_), ground-level ozone (O_3_), and nitrogen dioxide (NO_2_) measured at monitoring stations throughout Serbia. The collected daily concentrations of PM_2.5_, PM_10_, and NO_2_ were averaged per each participant’s period of interest: pre-pregnancy period (12 weeks preceding the first day of the last menstrual period), first trimester (first day of last menstrual period to 13 weeks and six days), and second trimester (14 weeks to 27 weeks and six days). For O_3_, the daily maximum 8 h average concentrations were used and subsequently averaged over each participant’s period of interest.

Given the substantial seasonal variations observed in the data, the LOWESS (Locally Weighted Scatterplot Smoothing) technique [31] was applied, utilizing average daily temperature data sourced from the Republic Hydrometeorological Service of Serbia [32] as a predictor variable. This was conducted using a Python (version 3.10) package (statsmodels). To ensure the validity of LOWESS regression, an additional package (sklearn; functions train_test_split and mean_squared_error) was used that automated the selection of the frac parameter, thereby determining the best fit.

After determining average concentrations for each participant’s periods of interest, their residential addresses were geocoded, and the Inverse Distance Weighting (IDW) method was used for spatial interpolation. For this purpose, we used ESRI ArcGIS Pro software (version 3.2.1). Concentrations of air pollutants from up to four monitoring stations within a 50 km radius were used to predict values at the participants’ residential addresses [33]. If participants reported relocating to another address during the study period, interpolated results were calculated for each address they resided in. The exposure levels were then assessed based on the duration of time spent at each particular address. To ensure the most accurate interpolation results with minimal root mean square error (RMSE), the power parameter was optimized using the built-in feature in ArcGIS Pro.

To account for the time spent indoors and outdoors, additional calculations were performed using Equation (1). This equation is based on concentrations of air pollutants weighted by the time spent indoors and outdoors.

E = (C × EF1/24) + (C × R × EF2/24)(1)

where E is the average exposure to the air pollutant for the period of interest (μg/m^3^); C is the average ambient concentration of the air pollutant for the same period (μg/m^3^); EF1 is the self-reported average time spent outdoors (h); EF2 is the self-reported average time spent indoors (h); and R is the ratio of indoor and outdoor air pollutant concentrations (I/O ratio). The I/O ratios used for this analysis are based on a study by Alonso-Blanco et al. [34]; for PM_2.5_, I/O of 0.18 for winter and 0.37 for summer was used; for PM_10_, I/O for 0.13 for winter and 0.18 for summer was used; for NO_2_, I/O of 1.19 for winter and 1.25 for summer was used; and for O3, I/O of 0.06 was used for both winter and summer. As these I/O ratios were reported in a study conducted in Spain, the seasons, same as in Serbia, refer to summer and winter months in the Northern Hemisphere.

### 2.5. Blood Sample Collection, DNA Extraction, and GSTM1 and GSTT1 Genotyping

Upon hospital admission, 5 mL blood samples were obtained from each participant and collected in EDTA tubes to prevent clotting. These samples were subsequently stored at −20 °C until DNA extraction and analysis could be conducted.

The DNA extraction from peripheral leukocytes was carried out using the salting-out method [35]. Following the extraction, *GSTM1* and *GSTT1* genotyping were conducted simultaneously using multiplex Polymerase Chain Reaction (PCR) [36]. In the PCR setup, 3 μL of the DNA isolates were combined with a 22 μL PCR mixture. This mixture contained the PCR Master Mix (EntiLink™, Elk Biotechnology, Wuhan, China), which provided the necessary components for the amplification reaction, including DNA polymerase, dNTPs, and reaction buffer. Specific primers targeting the *GSTM1* and *GSTT1* genes (Metabion, Planegg, Germany) were also added to the mixture. Sequences of primers used are reported in Table 1. The PCR was carried out under optimized cycling conditions, and the resulting products were then subjected to gel electrophoresis (8% polyacrylamide gels). After staining the gels with 0.5 μL/mL ethidium bromide, the presence or absence of the *GSTM1* and *GSTT1* genes was visualized under ultraviolet light. The *GSTM1*-null genotype was identified by the absence of a 215 bp band, which indicates the deletion of the *GSTM1* gene. Similarly, the *GSTT1*-null genotype was identified by the absence of a 480 bp band, indicating the deletion of the *GSTT1* gene. To prevent false-negative findings resulting from poor DNA quality or unsuccessful PCR, samples that showed deletion of both *GSTM1* and *GSTT1* were subjected to additional analysis using the primers for *β-globin* (Metabion, Planegg, Germany). If no *β-globin* was detected, the analysis was repeated until results could be obtained.

### 2.6. Statistical Analysis

Demographic and clinical data were summarized as categorical and continuous variables, expressed in terms of frequencies and percentages, as well as means and standard deviations, to which χ^2^ and *t*-test were employed to evaluate the differences between the case and control groups. Air pollutant exposure levels were presented as continuous variables, reported as means with standard deviations. Cohen’s d was employed to determine the effect size. Genotype distributions were reported as categorical variables and expressed in frequencies and percentages. Both air pollution exposure and genotype data were analyzed using Spearman’s rank correlation to assess associations. Logistic regression models, stratified by smoking status (non-smokers vs. smokers/former smokers), were applied to examine associations between GDM and genotypes or pollutant exposures, adjusting for potential confounders. Multiplicative interactions between genotypes and air pollutants were tested by incorporating interaction terms (genotype × pollutant) into logistic regression models. Statistical significance was defined as a two-tailed *p* < 0.05. To address unequal seasonal distributions among participants, all data concerning air pollution exposure were adjusted using inverse probability weighting, with the season of conception serving as a confounding variable. All analyses were conducted using R 4.3.0 Software (R Foundation for Statistical Computing, Vienna, Austria).

## 3. Results

### 3.1. Demographic and Clinical Characteristics

The total number of participants in the case group was 133, with a mean age of 32.4 ± 5.2, while the control group comprised 144 women with a mean age of 31.2 ± 4.8. The complete summary of demographic and clinical characteristics, along with the participants’ smoking habits, is presented in Table 2. Case and control groups exhibited strong similarities apart from the self-reported pre-pregnancy Body Mass Index (BMI). Women in the case group had higher BMI before pregnancy compared to those in the control group (t = 3.580; *p* < 0.001), with Cohen’s d indicating a medium effect size (d = 0.43).

### 3.2. The Associations of Exposure to Air Pollutants with Gestational Diabetes Mellitus

Analysis of the correlation between GDM and air pollutants during this period revealed a positive correlation for PM_2.5_ (ρ = 0.198; *p* < 0.001) with a medium effect size (d = 0.46); PM_10_ (ρ = 0.354; *p* < 0.001) with a large effect size (d = 0.80); and O_3_ (ρ = 0.259; *p* < 0.001) with a medium to large effect size (d = 0.74), suggesting higher exposures in the GDM group. In contrast, a marginal negative correlation was observed for NO_2_ (ρ = −0.093; *p* = 0.041), indicating higher exposures in the control group, although the effect size was negligible (d = 0.05).

During the first trimester, a positive correlation was found, a positive association was found for PM_10_ (ρ = 0.111; *p* = 0.015), with a small to medium effect size (d = 0.29) and O_3_ (ρ = 0.567; *p* < 0.001), with a very large effect size (d = 1.39), both pointing to higher exposures in the GDM group. However, NO_2_ exposures were higher in the control group, as shown by the negative correlation (ρ = −0.284; *p* < 0.001), and had a small to medium effect size (d = 0.29). No significant correlation was observed for PM_2.5_ (*p* = 0.208).

The second trimester exhibited patterns similar to the pre-pregnancy period, with higher exposures in the GDM group and PM_2.5_ (ρ = 0.208; *p* < 0.001), with a medium effect size (d = 0.57), PM_10_ (ρ = 0.344; *p* < 0.001), with a medium to large effect size (d = 0.77), and O_3_ (ρ = 0.393; *p* < 0.001), with a large effect size (d = 0.96). No significant correlations were observed for NO_2_ (*p* = 0.143).

**Figure 1:**
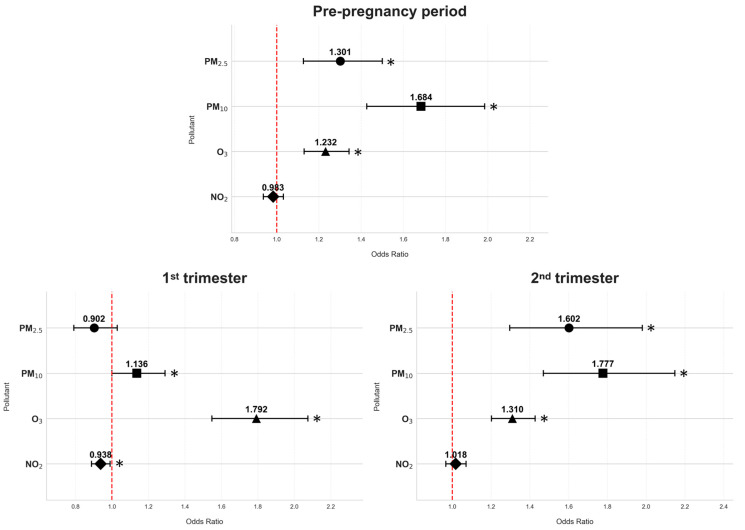
Odds ratios of air pollution exposure and gestational diabetes mellitus risk across various periods of interest in non-smoking women; * *p* < 0.05.

**Figure 2:**
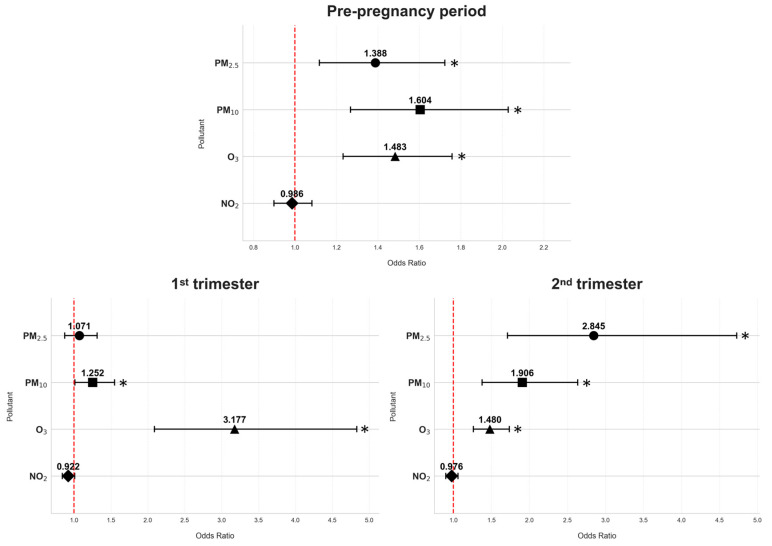
Odds ratios of air pollution exposure and gestational diabetes mellitus risk across various periods of interest in smoking/formerly smoking women; * *p* < 0.05.

Similar associations between GDM and exposure to air pollutants were observed when stratified by smoking status, adjusted for age and pre-pregnancy BMI, and weighted by season (Table 3, Figure 1 and Figure 2).

### 3.3. The Associations Between GSTM1-Null and GSTT1-Null Genotypes and Gestational Diabetes Mellitus Risk

**Figure 3:**
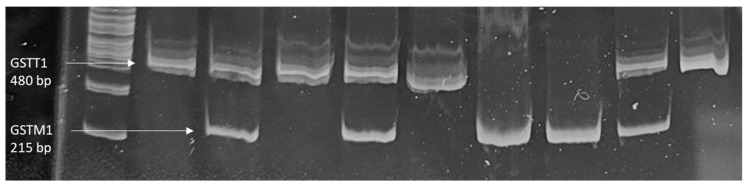
The visualization of *GSTM1* and *GSTT1* on ethidium bromide-stained polyacrylamide gels under ultraviolet light.

Figure 3 presents the visualization of *GSTM1* and *GSTT1* on polyacrylamide gels under ultraviolet light. The absence of the 215 bp band indicated GSTM1 deletion, and the absence of the 480 bp band indicated *GSTT1* deletion.

The *GSTM1*-null genotype had an overall prevalence of 48.4% (134 participants) in the study population. Among women with GDM, the *GSTM1*-null genotype was identified in just under half of the participants (65 individuals; 48.9%), a frequency similar to that observed in the control group (69 individuals; 47.9%). Meanwhile, the *GSTT1*-null genotype had an overall prevalence of 19.9% (55 participants). The frequency of the *GSTT1*-null genotype was marginally higher in women with GDM (28 individuals; 21.1%) compared to those without GDM (27 individuals; 18.8%). Among all participants, the deletion of both *GSTM1* and *GSTT1* was observed in 26 individuals (9.4%). This included 13 participants from the case group (9.8%) and 13 from the control group (9.0%).

Analysis of the associations between GDM with the *GSTM1*-null genotype revealed no statistically significant correlation (ρ = −0.010; *p* = 0.867). Similarly, the correlation between GDM with the *GSTT1*-null genotype was also non-significant (ρ = −0.056; *p* = 0.353). Furthermore, after adjusting for covariates of age and pre-pregnancy BMI, and stratified by smoking status, no significant associations were observed between GDM and either the *GSTM1*-null or *GSTT1*-null genotypes (Table 4).

### 3.4. GSTM1/GSTT1–Air Pollutant Interaction and the Risk of Gestational Diabetes Mellitus

**Figure 4:**
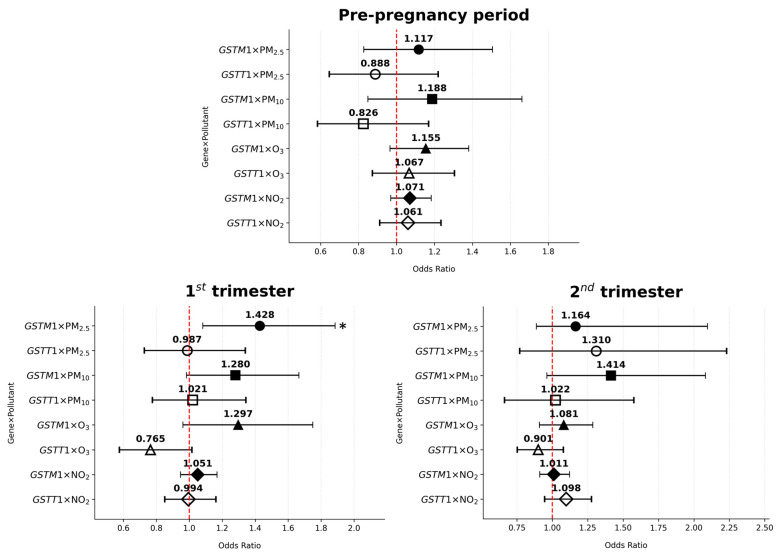
Odds ratios of *GSTM1*/*GSTT1–*pollutant interactions and gestational diabetes mellitus risk across various periods of interest in non-smoking women; * *p* < 0.05.

**Figure 5:**
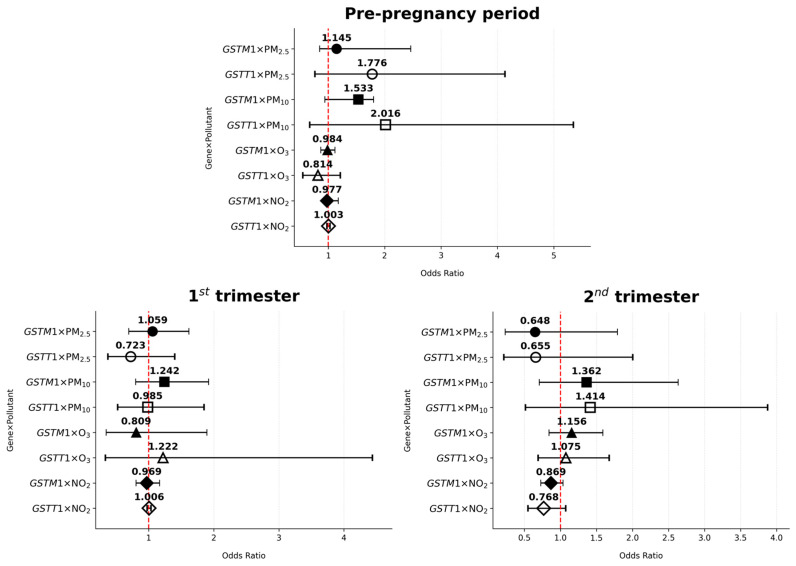
Odds ratios of *GSTM1*/*GSTT1–*pollutant interactions and gestational diabetes mellitus risk across various periods of interest in smoking/formerly smoking women.

The associations between *GSTM1-/GSTT1*-null genotype–air pollutant interaction and the risk of GDM are presented in Table 5 and Figure 4 and Figure 5. The gene–environment analysis revealed one significant association: PM_2.5_ exposure in the first trimester elevated GDM risk among non-smoking *GSTM1*-null women (OR = 1.428, 95% CI: 1.082–1.885, *p* = 0.012).

## 4. Discussion

While global maternal-child health strategies have effectively targeted modifiable metabolic risks like obesity and poor nutrition, growing evidence suggests that environmental pollutants may represent additional risk factors for GDM. However, not all women exposed to elevated pollution levels develop illness, just as some with lower exposure may still experience health consequences. This discrepancy challenges the dominant role of air pollution and suggests additional factors in disease development, including genetic differences, particularly in genes involved in environmental pollutant detoxification.

The results from our study indicate that exposure to air pollutants during different periods may be a significant risk factor for GDM. In non-smoking women, higher exposure to PM_2.5_ during the pre-pregnancy period (OR = 1.301; 95% CI: 1.127–1.501) and second trimester (OR = 1.602; 95% CI: 1.296–1.980) was significantly associated with higher GDM risk, much like in smoking/formerly smoking women (pre-pregnancy: OR = 1.338; 95% CI: 1.118–1.723; second trimester: OR = 2.845; 95% CI: 1.712–4.728). Similarly, in non-smoking women, PM_10_ exposure showed increased risks in all periods, including pre-pregnancy (OR = 1.684; 95% CI: 1.427–1.986), first trimester (OR = 1.136; 95% CI: 1.000–1.291), and second trimester (OR = 1.777; 95% CI: 1.470–2.148), and the results were similar for smoking/formerly smoking women (pre-pregnancy: OR = 1.604; 95% CI: 1.268–2.029; first trimester: OR = 1.252; 95% CI: 1.012–1.548; and second trimester: OR = 1.906; 95% CI: 1.378–2.636). For gaseous pollutants, in non-smoking women, O_3_ had significant positive associations with GDM risk in all periods of interest: pre-pregnancy (OR = 1.232; 95% CI: 1.130–1.343), first trimester (OR = 1.792; 95% CI: 1.548–2.075), and second trimester (OR = 1.310; 95% CI: 1.202–1.427); the associations in smoking/formerly smoking women followed the same patterns (pre-pregnancy: OR = 1.438; 95% CI: 1.232–2.758; first trimester: OR = 3.177; 95% CI: 2.090–4.831; and second trimester: OR = 1.480; 95% CI: 1.262–1.736). However, it is important to note that, for smoking/formerly smoking women, the CIs for O_3_ in the first trimester and PM_2.5_ in the second appear quite wide, pointing to a level of uncertainty. On the other hand, NO_2_ exposure in non-smoking women during the first trimester had an inverse relationship with GDM risk (OR = 0.938; 95% CI: 0.888–0.990).

These findings somewhat align with broader epidemiological evidence. A meta-analysis by Ren et al. [37], which included results of 35 studies, reported significant associations between PM_2.5_ exposure and GDM risk during preconception (OR = 1.09; 95% CI: 1.05–1.14), first trimester (OR = 1.05; 95% CI: 1.01–1.08), and second trimester (OR = 1.07; 95% CI: 1.03–1.10). The analysis of PM_10_ has shown less consistent results, showing significance only in the preconception period (OR = 1.09; 95% CI: 1.00–1.19) and no notable associations in later periods. Furthermore, the authors found no significant correlations between GDM risk and exposure to O_3_ or NO_3_ in any trimester, differing from our results for O_3_ and the inverse trend for NO_3_. These discrepancies may be due to differences in study design, exposure assessment methods, or population-specific factors. But the intriguing inverse correlation for NO_2_ observed in our study remains entirely unclear. One potential explanation lies in the inverse relationship between NO_2_ and O_3_, driven by the atmospheric NO, NO_2_, and O_3_ cycle. Under typical conditions, when exposed to sunlight, NO_2_ breaks down into NO and O^−^, which then react with O_2_ to form O_3_. At night, this reaction reverses. However, in high-traffic areas, such as near highways, heavy NO emissions tend to suppress O_3_ levels because excess NO reacts with O_3_, resulting in NO_2_ and O_2_. Conversely, in suburban or low-traffic regions where NO is scarce, O_3_ accumulates while NO_2_ remains relatively low [38]. Since the results from our study point to the very strong association between O_3_ and GDM risk, the observed inverse correlation with NO_2_ may simply reflect this chemical antagonism, or an artefact, rather than a true biological effect.

The explanation for the potential negative effects of air pollution could be in the pathophysiological mechanisms it activates. These processes start by inhaling small particles, small enough to cross the lung–blood barrier and enter the bloodstream. The particles can then be disseminated further to distant tissues and cause the creation of ROS, consequently leading to oxidative stress and cell damage. In GDM, this is particularly important in pancreatic β-cells, as they are highly sensitive to oxidative stress, and this damage inhibits their ability to secrete insulin [5]. Apart from directly affecting cells, the indicated mechanisms are indirectly giving rise to inflammatory processes by causing macrophages and neutrophils to secrete proinflammatory cytokines. Simultaneously, particles larger in size that cannot cross the lung–blood barrier, as well as gases, cause an inflammatory response in the lungs. This inflammatory response, however, is not restricted to the lungs, as these cytokines are also present in the blood after exposure, making a state of chronic low-grade inflammation [39,40]. This chronic low-grade inflammation can interfere with the insulin signaling pathway, ultimately leading to insulin resistance. Both of the described factors, damage to pancreatic β-cells and insulin resistance, are significant in GDM development [5,41].

Our findings show no significant association between the *GSTM1*-null or *GSTT1*-null genotype and GDM susceptibility. These results are consistent with prior work by Orhan et al. [28], which also found no significant correlations. Based on our findings, the lack of associations of explored genes and the previously mentioned strong associations with exposure to air pollution indicate that environmental factors may outweigh GST defense mechanisms and lead to the development of GDM.

However, the lack of association between these genetic deletions and GDM could still not be completely ruled out as a potential variable in GDM development. Even when these genes are present, their expression could be changed by epigenetic modification (e.g., DNA methylation or histone modifications), thereby altering their role in detoxification [42,43]. On the other hand, if deletion is present and genes are not functional, other genes belonging to the same superfamily could be upregulated, which compensates for their loss. As reported in a study by Bhattacharjee et al. [44], the absence of *GSTM1* activity would lead to overexpression of *GSTM2*, thereby effectively maintaining detoxification capacity.

The results of our research have demonstrated a significant association between the interaction of the analyzed genotypesand air pollution with the occurrence of GDM. This interaction points to a higher risk of GDM in non-smoking women with the *GSTM1-*null genotype who were exposed to higher levels of PM_2.5_ during the first trimester. However, no such interaction has been observed in smoking or formerly smoking women. One explanation for this difference is due to the already increased baseline risk in women who smoke or have smoked before pregnancy [45]. In these women, the effects of smoking and PM2.5 exposure may dominate the metabolic pathways leading to GDM, obscuring the additional contribution of the *GSTM1*-null genotype, thereby rendering it biologically negligible. At the same time, non-smoking women have a lower baseline risk, so the effect of the interaction between PM_2.5_ and *GSTM1*-null genotype becomes more pronounced.

Another important aspect of this result is that, among all the analyzed air pollutants, the interaction was observed between PM_2.5_ and the *GSTM1*-null genotype. The potential explanation for this particular interaction could be in the function of GST. As previously mentioned, these enzymes play a role in the detoxification of environmental pollutants, primarily carcinogens, among which the most commonly found in air pollution are polycyclic aromatic hydrocarbons (PAHs) [46]. The predominant exposure (70–90%) to airborne PAHs is through their carriers–particulate matter, most notably PM_2.5_ [47]. This suggests that the interaction between air pollution and *GST* genes is entirely dependent on the constituents of air pollutants, primarily particulate matter. Therefore, the chemical speciation of particulate matter is essential for mechanistic conclusions.

Unfortunately, to the best of our knowledge, at this time, there are no published research papers on the influence of this interaction on the development of GDM. Indicated interaction has mostly been the object of research focusing on the effects on respiratory illnesses, particularly asthma and allergies [48,49]. These authors suggest that the interaction is likely; however, due to significant deviations in methodologies and populations, potential gene–gene interactions, or the impact of other environmental factors, it is challenging to come to a definitive conclusion. On the topic of glucoregulation disorders, research performed by Kim et al. [50], conducted on a population of elderly Koreans, suggested that the increased risk of developing insulin resistance is found in people carrying *GSTM1*-null and *GSTT1*-null genotypes that have been exposed to higher levels of PM_2.5_, O_3_, and NO_2_. Regarding disorders developed during pregnancy, only one research paper has been published at this time, and the results demonstrate the presence of interaction between *GST* genes and air pollution [51]. The authors of this paper examined the effects of this interaction on the risk of preterm birth and concluded that women carrying the *GSTM1*-null genotype incur a higher risk of preterm birth if exposed to higher levels of PM_10_ during the third trimester. Our research excludes the third trimester due to GDM being diagnosed earlier in pregnancy.

Studies that focus on gene–environment interactions and GDM are few and far between. Air pollution as an environmental factor has been the subject of a handful of these studies. Originally, Yang et al. [52] demonstrated that the polymorphisms of the *IGF2BP2* gene in women exposed to higher levels of PM_2.5_ and O_3_ increased the risk of developing GDM. Subsequently, a study by Huang et al. [53] showed that the presence of polymorphisms of genes *LINGO2* and *GLIS3* leads to an increased risk of developing GDM in women exposed to PM_2.5_ and O_3_.

Lastly, we would like to point out several strengths and limitations of our study. First, to the best of our knowledge, this is the first study to investigate the role of the interaction of air pollution and *GST* genes on the development of GDM. Also, this is the first study focusing on air pollution as a risk factor for GDM in Serbia, a country where air pollutant concentrations are among the highest in Europe [54]. Second, the air pollution exposure assessment method we used could be considered both a strength and a limitation. In our analysis, we focused on individual factors not often taken into consideration, such as residential mobility and time spent indoors. On the other hand, the I/O ratios used to determine indoor air quality can vary greatly depending on the activities at home. Furthermore, these I/O ratios were derived from urban environments and may not accurately represent pollution infiltration patterns in other settings. Third, although we used different techniques to account for seasonal variability as a confounding factor, most of our participants were recruited during seasons when peaks in air pollutant concentrations are most prevalent. Fourth, in this study, we did not determine the constituents of particulate matter (e.g., PAHs) since these are not part of the routinely reported data. Fifth, this study did not assess dietary intake of antioxidants, which may influence the overall antioxidant capacity and, therefore, our findings. Sixth, although the exclusion of women under the age of 18 adheres to the strong ethical framework, and the exclusion of highly mobile women provides the strong scientific rigor in exposure assessment, these criteria may introduce selection bias. Lastly, although we presented several significant results, this research had a relatively small sample size, as is often the case in single-center studies, particularly those focusing on multiple factors. This is particularly notable in the subgroup of smoking/formerly smoking women with the *GSTT1*-null genotype, where the wider confidence intervals indicate a degree of uncertainty in the findings. Additional studies with more participants and different populations are needed to expand our knowledge of this complex disorder.

## 5. Conclusions

The findings of this study add to growing evidence that exposure to air pollution across critical time windows plays a major role in GDM development. While we found no association between *GSTM1-/GSTT1*-null genotypes and GDM risk, our interaction analysis revealed an important conclusion: Non-smoking women with the *GSTM1*-null genotype demonstrated significantly elevated risk of GDM when exposed to PM_2.5_ during early pregnancy. This gene–environment interaction indicates the particular susceptibility of this subpopulation to air pollution-induced metabolic disruption during a sensitive developmental period.

While studies regarding gene–environment interactions remain valuable and continue to identify the most vulnerable individuals, our findings emphasize the need for stronger implementations of air pollution control and effect mitigation strategies. These strategies should be structured across multiple tiers: (1) strengthening policy-level measures to reduce air pollutant emissions, (2) implementing personal-level protection behaviors in highly polluted areas (e.g., reducing time spent in polluted environments, wearing facemasks, and using air purifiers at home), encouraged by healthcare personnel, and (3) strengthening defenses through antioxidant-rich diets. Together, these strategies can reduce the risk of GDM across all genetic backgrounds until further studies refine precision prevention.

## Figures and Tables

**Table 1:** PCR primer sequences used in the analysis.

Gene	Gene Accession Number	Primer Sequence
*GSTM1*	NC_000001.11	5′-GTTGGGCTCAAATATACGGTGG-3′ 5′-GAACTCCCTGAAAAGCTAAAGC-3′
*GSTT1*	NT_187633.1	5′-TTCCTTACTGGTCCTCACATCT-3′ 5′-TCACCGGATCATGGCCAGCA-3′
*β-globin*	NC_000011.10	5′-ACACAACTGTGTTCAACTAGC-3′ 5′-CAACTTCATCCACGTTCACC-3′

**Table 2:** Demographic and clinical characteristics, and smoking habits of pregnant women with and without gestational diabetes mellitus (N = 277).

Characteristic	Case Group (n = 133)	Control Group (n = 144)	Total (n = 277)	χ^2^/t Value	*p* Value
Age (years) n (%)					
<30	41 (30.8)	48 (33.3)	89 (32.1)	0.199 *	0.655 *
≥30	92 (69.2)	96 (66.7)	188 (67.9)		
Weight gain during pregnancy (kg) n (%)					
<16	94 (70.7)	90 (62.5)	184 (66.4)	2.073 *	0.150 *
≥16	39 (29.3)	54 (37.5)	93 (33.6)		
Parity n (%)					
1	90 (67.7)	78 (54.2)	168 (61.0)	5.305 *	0.070 *
2	34 (25.5)	53 (36.8)	78 (28.1)		
≥3	9 (6.8)	13 (9.0)	22 (7.9)		
Smoking status n (%)					
No	100 (75.2)	101 (70.1)	201 (72.6)	1.940 *	0.379 *
Yes	10 (7.5)	18 (12.5)	28 (10.1)		
Former smoker	23 (17.3)	25 (17.4)	48 (17.3)		
Pre-pregnancy BMI (kg/m^2^) Mean (SD)	24.60 (4.67)	22.60 (3.41)	23.41 (4.09)	3.580 ^†^	<0.001 ^†^

* χ^2^ test; ^†^ *t*-test.

**Table 3:** Associations between air pollutant exposure and the gestational diabetes mellitus risk across various periods of interest, stratified by smoking status (N = 277).

	Case Group Mean (SD)	Control Group Mean (SD)	β *	*p* Value *	OR *	95% CI *
Non-smokers	N = 100	N = 101				
PM_2.5_						
Pre-pregnancy	7.22 (1.98)	6.51 (1.21)	0.263	<0.001	1.301	1.127–1.501
1st trimester	6.56 (2.19)	6.78 (1.23)	−0.103	0.126	0.902	0.791–1.029
2nd trimester	5.01 (1.84)	4.31 (0.75)	0.471	<0.001	1.602	1.296–1.980
PM_10_						
Pre-pregnancy	8.09 (1.95)	6.84 (1.22)	0.521	<0.001	1.684	1.427–1.986
1st trimester	7.51 (2.13)	7.07 (1.36)	0.127	0.050	1.136	1.000–1.291
2nd trimester	6.70 (1.91)	5.60 (0.97)	0.575	<0.001	1.777	1.470–2.148
O_3_						
Pre-pregnancy	7.88 (3.95)	6.14 (2.02)	0.209	<0.001	1.232	1.130–1.343
1st trimester	8.03 (3.69)	4.79 (1.60)	0.583	<0.001	1.792	1.548–2.075
2nd trimester	9.60 (3.72)	7.06 (2.46)	0.270	<0.001	1.310	1.202–1.427
NO_2_						
Pre-pregnancy	31.78 (5.65)	32.10 (3.45)	−0.017	0.492	0.983	0.937–1.032
1st trimester	29.87 (5.12)	31.10 (3.49)	−0.064	0.020	0.938	0.888–0.990
2nd trimester	28.04 (4.97)	27.94 (3.79)	0.017	0.506	1.018	0.967–1.071
Smokers/Former smokers	N = 33	N = 43				
PM_2.5_						
Pre-pregnancy	7.87 (2.64)	6.69 (1.37)	0.328	0.003	1.388	1.118–1.723
1st trimester	7.13 (2.45)	6.82 (1.25)	0.068	0.512	1.071	0.873–1.313
2nd trimester	5.20 (1.46)	4.34 (0.60)	1.046	<0.001	2.845	1.712–4.728
PM_10_						
Pre-pregnancy	8.57 (2.29)	6.89 (1.47)	0.473	<0.001	1.604	1.268–2.029
1st trimester	8.01 (2.19)	7.20 (1.43)	0.225	0.038	1.252	1.012–1.548
2nd trimester	6.80 (1.31)	5.68 (1.20)	0.645	<0.001	1.906	1.378–2.636
O_3_						
Pre-pregnancy	8.53 (3.15)	6.25 (1.69)	0.394	<0.001	1.483	1.232–1.758
1st trimester	8.41 (2.23)	4.81 (1.52)	1.156	<0.001	3.177	2.090–4.831
2nd trimester	10.48 (3.59)	6.94 (2.44)	0.392	<0.001	1.480	1.262–1.736
NO_2_						
Pre-pregnancy	30.66 (3.51)	31.78 (4.35)	−0.014	0.467	0.986	0.899–1.082
1st trimester	28.58 (3.54)	30.05 (4.25)	−0.082	0.081	0.922	0.841–1.010
2nd trimester	26.50 (4.96)	27.06 (4.23)	0.025	0.559	0.976	0.899–1.059

* Adjusted for age and pre-pregnancy BMI, and weighted by the season of conception.

**Table 4:** The associations between *GSTM1*- and *GSTT1*-null genotypes and gestational diabetes mellitus risk, stratified by smoking status (N = 277).

Genotype	Case Group N (%)	Control Group N (%)	β *	*p* Value *	OR *	95% CI *
Non-smokers	N = 100	N = 101				
*GSTM1* present	50 (50.0)	57 (56.4)	0.192	0.514	1.212	0.681–2.156
*GSTM1*-null genotype	50 (50.0)	44 (43.6)				
*GSTT1* present	80 (80.0)	83 (82.2)	0.217	0.558	1.242	0.601–2.567
*GSTT1*-null genotype	20 (20.0)	18 (17.8)				
Smokers/Former smokers	N = 33	N = 43				
*GSTM1* present	18 (54.5)	18 (41.9)	−0.456	0.350	0.634	0.243–1.651
*GSTM1*-null genotype	15 (45.5)	25 (58.1)				
*GSTT1* present	25 (75.8)	34 (79.1)	0.020	0.973	1.020	0.322–3.227
*GSTT1*-null genotype	8 (24.2)	9 (20.9)				

* Adjusted for age and pre-pregnancy BMI.

**Table 5:** The associations between *GSTM1*/*GSTT1–*air pollutant interaction and the risk of gestational diabetes mellitus across various periods of interest, stratified by smoking status.

Period	Pollutant	Interaction	β *	*p* Value *	OR *	95% CI *
Non-smokers						
Pre-pregnancy	PM_2.5_	*GSTM1* × PM_2.5_	0.110	0.469	1.117	0.828–1.506
		*GSTT1* × PM_2.5_	−0.119	0.464	0.888	0.646–1.220
	PM_10_	*GSTM1* × PM_10_	0.172	0.315	1.188	0.849–1.661
		*GSTT1* × PM_10_	−0.191	0.280	0.826	0.584–1.169
	O_3_	*GSTM1* × O_3_	0.144	0.115	1.155	0.966–1.381
		*GSTT1* × O_3_	0.065	0.526	1.067	0.873–1.306
	NO_2_	*GSTM1* × NO_2_	0.069	0.175	1.071	0.970–1.183
		*GSTT1* × NO_2_	0.059	0.442	1.061	0.912–1.235
1st trimester	PM_2.5_	*GSTM1* × PM_2.5_	0.356	0.012	1.428	1.082–1.885
		*GSTT1* × PM_2.5_	−0.013	0.934	0.987	0.727–1.340
	PM_10_	*GSTM1* × PM_10_	0.247	0.067	1.280	0.983–1.666
		*GSTT1* × PM_10_	0.021	0.881	1.021	0.776–1.344
	O_3_	*GSTM1* × O_3_	0.260	0.090	1.297	0.961–1.750
		*GSTT1* × O_3_	−0.268	0.064	0.765	0.576–1.016
	NO_2_	*GSTM1* × NO_2_	0.050	0.350	1.051	0.947–1.168
		*GSTT1* × NO_2_	−0.006	0.940	0.994	0.851–1.161
2nd trimester	PM_2.5_	*GSTM1* × PM_2.5_	0.310	0.157	1.164	0.887–2.096
		*GSTT1* × PM_2.5_	0.270	0.319	1.310	0.770–2.231
	PM_10_	*GSTM1* × PM_10_	0.347	0.079	1.414	0.961–2.082
		*GSTT1* × PM_10_	0.021	0.923	1.022	0.662–1.576
	O_3_	*GSTM1* × O_3_	0.077	0.383	1.081	0.908–1.286
		*GSTT1* × O_3_	−0.105	0.255	0.901	0.752–1.078
	NO_2_	*GSTM1* × NO_2_	0.011	0.840	1.011	0.911–1.122
		*GSTT1* × NO_2_	0.093	0.221	1.098	0.945–1.276
Smokers/Former smokers						
Pre-pregnancy	PM_2.5_	*GSTM1* × PM_2.5_	0.432	0.069	1.145	0.847–2.464
		*GSTT1* × PM_2.5_	0.574	0.183	1.776	0.763–4.136
	PM_10_	*GSTM1* × PM_10_	0.427	0.089	1.533	0.937–1.804
		*GSTT1* × PM_10_	0.701	0.159	2.016	0.670–5.348
	O_3_	*GSTM1* × O_3_	−0.016	0.803	0.984	0.865–1.119
		*GSTT1* × O_3_	−0.206	0.312	0.814	0.546–1.213
	NO_2_	*GSTM1* × NO_2_	−0.023	0.809	0.977	0.911–1.177
		*GSTT1* × NO_2_	−0.003	0.845	1.003	0.976–1.030
1st trimester	PM_2.5_	*GSTM1* × PM_2.5_	0.057	0.792	1.059	0.693–1.618
		*GSTT1* × PM_2.5_	−0.324	0.336	0.723	0.374–1.399
	PM_10_	*GSTM1* × PM_10_	0.206	0.332	1.242	0.801–1.922
		*GSTT1* × PM_10_	−0.015	0.963	0.985	0.524–1.851
	O_3_	*GSTM1* × O_3_	−0.212	0.625	0.809	0.346–1.893
		*GSTT1* × O_3_	0.200	0.761	1.222	0.336–4.436
	NO_2_	*GSTM1* × NO_2_	−0.031	0.742	0.969	0.805–1.167
		*GSTT1* × NO_2_	0.006	0.708	1.006	0.977–1.035
2nd trimester	PM_2.5_	*GSTM1* × PM_2.5_	−0.463	0.402	0.648	0.233–1.791
		*GSTT1* × PM_2.5_	−0.432	0.458	0.655	0.214–2.002
	PM_10_	*GSTM1* × PM_10_	0.309	0.358	1.362	0.705–2.633
		*GSTT1* × PM_10_	0.347	0.500	1.414	0.516–3.874
	O_3_	*GSTM1* × O_3_	0.145	0.369	1.156	0.842–1.588
		*GSTT1* × O_3_	0.072	0.750	1.075	0.689–1.676
	NO_2_	*GSTM1* × NO_2_	−0.141	0.116	0.869	0.728–1.036
		*GSTT1* × NO_2_	−0.264	0.121	0.768	0.550–1.073

* Adjusted for age and pre-pregnancy BMI, and weighted by the season of conception.

## Data Availability

The data presented in this study are available on request from the corresponding author due to patient confidentiality.

## Abbreviations

The following abbreviations are used in this manuscript:

GDM	Gestational diabetes mellitus
ROS	Reactive oxygen species
GSTs	Glutathione transferases
GSH	Glutathione
Nrf2	Nuclear factor erythroid 2-related factor 2
Keap1	Kelch-like ECH-associated protein 1
ARE	Antioxidant response element
GSTM1	Glutathione S-transferase mu 1
GSTT1	Glutathione S-transferase theta 1
T2DM	Diabetes mellitus type 2
